# Size Effect on Shear Behaviors of 3D-Printed Soft Rock-like Materials Under Different Shear Velocities

**DOI:** 10.3390/ma17246180

**Published:** 2024-12-18

**Authors:** Chaolei Wu, Lishuai Jiang, Yang Zhao, Qi Wu, Yiming Yang, Xiaohan Peng, Pimao Li

**Affiliations:** 1 College of Energy and Mining Engineering, Shandong University of Science and Technology, Qingdao 266590, China; m18881007624@163.com (C.W.); lsjiang@sdust.edu.cn (L.J.); wuqi2628693253@163.com (Q.W.); morningdayym@163.com (Y.Y.); asperata0516@163.com (X.P.); lipimao9908@163.com (P.L.); 2 State Key Laboratory of Mining Disaster Prevention and Control, Shandong University of Science and Technology, Qingdao 266590, China

**Keywords:** rock-like material, 3D printing, shear properties, size effect, failure pattern

## Abstract

The shear failure of rock masses is one of the primary causes of underground engineering instability. The shear mechanical behavior of rocks at different sizes is of great significance for studying the shear failure pattern of engineering rock masses. However, due to the presence of various joints and defects in natural rocks, the obtained rock specimens exhibit significant discreteness, making it difficult to customize specimen sizes for size effect studies. In recent years, 3D printing (3DP) technology has gained widespread application in rock mechanics tests due to its high printing precision and ability to form specimens in a single step with minimal discreteness. Among these, specimens prepared using sand-powder 3DP exhibit elastoplastic mechanical characteristics similar to those of natural rocks. Therefore, this study utilized sand-powder 3DP to prepare rock-like specimens of four different sizes and conducted compression shear tests under three different shear velocities. The shear strength, shear strain, and wear of the shear surfaces were analyzed as functions of specimen size and shear velocities. The results indicate that under the same shear velocity, the shear strength of the specimens is negatively correlated with specimen size. The peak shear strain is generally unaffected by shear velocities, but it increases initially and then decreases with increasing specimen size. As specimen size increases, the degree of specimen damage intensifies, and larger specimens are more prone to developing derived fractures. This study broadens the application of sand-powder 3DP technology in investigating the shear mechanical properties of soft rocks, offering novel insights into the study of size effects in rock mechanics. However, the current research does not encompass tests on 3D-printed rock specimens with varying printing directions, nor does it delve into the role of fractures in size effect analyses. Future investigations will aim to address these limitations, thereby advancing the applicability of 3D printing technology in rock mechanics research and enhancing its contributions to the field.

## 1. Introduction

The study of rock shear mechanics is crucial for ensuring the stability and safety of engineering structures. In practical engineering, the shear behavior of rock directly affects the bearing capacity and deformation characteristics of rock masses, posing significant threats to the overall safety and stability of the projects. Soft rock is defined as a rock mass capable of undergoing significant deformation under applied engineering forces, with uniaxial compressive strength typically ranging from 0.5 to 25 MPa [1]. In mining operations, rock shear failure, particularly in soft rock formations, severely constrains the safe and efficient production of coal mines, endangering both lives and property. Moreover, different types of rocks and differences in geological conditions make the study of shear behavior highly specific and complex. For instance, in areas with frequent seismic activity or rock bursts, changes in in situ stress can significantly influence rock shear behavior [2]. Therefore, accurately understanding and predicting rock shear behavior is of great importance for effectively preventing engineering accidents and ensuring the long-term stability of engineering structures [3].

As a quasi-brittle material, natural rock exhibits a significant size effect, where its mechanical or physical properties vary with the specimen size [4]. This phenomenon is particularly pronounced in practical engineering applications, where rocks of different sizes show notable differences in load-bearing capacity, deformation characteristics, and failure patterns [5]. Systematic studies on the size effect of rocks can reveal the patterns of how mechanical parameters change with size, thereby providing more accurate parameters for engineering design. This is directly beneficial for improving the reliability and safety of rock engineering design. Numerous scholars have studied the size effect of various types of rocks through uniaxial compression tests, reaching relatively consistent conclusions [6,7,8]. Bandis et al. [9] demonstrated that rocks of different sizes exhibit significant differences in their shear mechanical properties. However, despite extensive research by scholars worldwide, there is no unified understanding of the size effect under shear conditions [10]. Furthermore, under varying engineering geological conditions, the shear velocity experienced by rocks varies significantly, and the deformation and failure characteristics of rocks exhibit clear velocity dependency [11,12]. Therefore, investigating the size effects of rocks at different shear velocities and exploring the patterns of how mechanical parameters change with size is of great importance and practical value for predicting the failure and instability morphology of rock masses in actual engineering projects.

Scholars have conducted extensive research on the shear–size effect of rocks. Pan et al. [13] analyzed the shear failure size effect of rocks based on gradient plasticity theory. Huang et al. [14] found that, under the same normal stress, the shear strength of structural surface specimens was not significantly affected by size changes. Wang et al. [15] used the discrete element method to establish rough discrete joint network models of different sizes and conducted numerical simulation tests, which showed that the shear strength of the models exhibited a negative size effect. Bahaaddini et al. [16] summarized the research results of various scholars, categorizing the size effect into positive size effects, negative size effects, and no size effects, further revealing the diversity of the shear size effect. Barton [17] suggested that sampling bias might affect the accuracy of some size effect conclusions, pointing out that the difficulties in sampling could hinder the research on shear size effects. These studies indicate that the size effect of rock shear behavior has been a focal point in the field of rock mechanics. However, due to the complexity of factors influencing shear behavior and the discreteness of specimens, studying the size effect of shear behavior faces numerous challenges. For instance, in size effect research, natural sampling methods encounter problems such as high specimen discreteness, difficulty in sampling, and challenges in achieving customizable specimen sizes. These factors severely constrain the systematic study of the size effect and lead to the discreteness and uncertainty of research results. Therefore, overcoming these difficulties and finding a method to precisely control the size and shape of specimens has become a key issue in the study of shear size effects.

**Figure 1:**
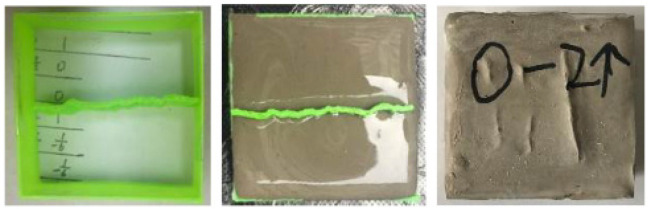
Flow chart of rock-like specimen prepared by PLA mold [23].

In recent years, the rapidly developing 3D printing (3DP) technology has been widely applied in fields such as aerospace, medicine, and robotics due to its advantages in producing high-precision, customizable models and short preparation cycles. It has also provided new methods for rock mechanics research [18,19,20]. Xiong et al. [21] conducted a series of direct shear tests on joints using 3DP technology, revealing the feasibility of applying 3DP technology in shear mechanical tests. Jiang et al. [22] and Wang et al. [23] used Fused Deposition Modeling (FDM) to prepare Polylactic Acid (PLA) joint models with different Joint Roughness Coefficient (JRC) values, then used these PLA models to cast structural surface specimens with varying roughnesses for shear tests. These scholars have conducted preliminary explorations on the applicability of 3DP technology in shear tests. However, limited by the printing technology and materials, they primarily used 3DP technology for mold making, where the shear test objects were rock-like specimens cast from the molds (see Figure 1) rather than 3DP one-piece specimens. The main reason for this limitation was the significant difference in mechanical properties between earlier 3DP materials and rocks. Extensive tests have validated that sand-powder 3DP soft rock, using sand-powder as the base material, has elastoplastic mechanical characteristics very similar to natural coal, mudstone, and sandstone, making it a promising material for manufacturing rock-like specimens [24,25,26,27]. Sand-powder 3DP soft rock is prepared using Binder Jetting Technology (BJT), which has a molding principle similar to the formation process of sedimentary rocks. This technology allows for the flexible customization of various sizes and geometric shapes of specimens according to test needs in a short time, greatly enhancing test efficiency. Additionally, this process can precisely control specimen variables, enabling parallel tests of different factors, thus increasing the reliability and comparability of results [28,29].

Some scholars have utilized the advantage of 3DP technology in quickly preparing specimens of various sizes to conduct preliminary explorations on the size effect of sand-powder 3DP soft rock. Wang et al. [30] used BJT technology to study the uniaxial compression size effect of sand-powder 3DP soft rock, demonstrating the feasibility of BJT technology in size effect research. Luo et al. [31] studied the size effect of sand-powder 3DP fractured specimens through uniaxial compression tests, finding that the uniaxial compressive strength of the specimens exhibited a significant negative size effect. Wang et al. [32] combined uniaxial compression tests with Digital Image Correlation (DIC) systems to prove that sand-powder 3DP soft rock has a size effect similar to that of some natural rock mass. Thus, the uniaxial compression size effect of sand-powder 3DP soft rock has been fully verified, but its shear mechanical behavior remains to be studied.

Therefore, in this study, four kinds of sand-powder 3D-printed soft rock samples with different sizes were prepared by BJT. Shear tests were conducted under constant normal stress conditions to analyze the impact of specimen size on shear strength and failure patterns. The results were compared with the shear size effect patterns of some natural rocks, verifying the effectiveness of sand-powder 3DP soft rock in simulating the physical properties of coal and other soft rocks. The findings revealed the shear mechanical properties of sand-powder 3DP soft rock, further enriching its applicability in rock shear mechanics research. This study lays the foundation for subsequent shear tests on fractured rocks using this method, offering new insights and possibilities for the broad application of sand-powder 3DP technology in rock mechanics.

## 2. Specimen Preparation and Test Design

### 2.1. Preparation of Sand-Powder 3D Printed Specimens

**Figure 2:**
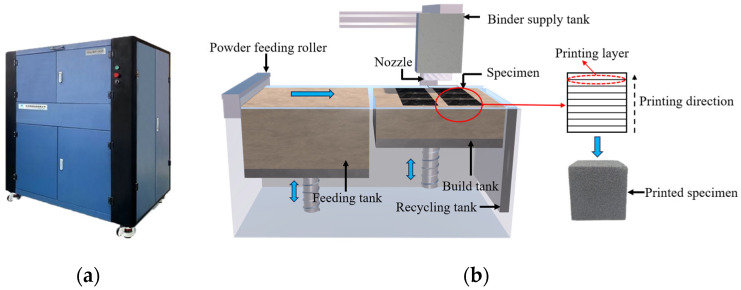
A 3D printer and specimen preparation flow chart: (**a**) Easy3DP-S450 3D printer; (**b**) BJT process for specimen preparation.

**Figure 3:**
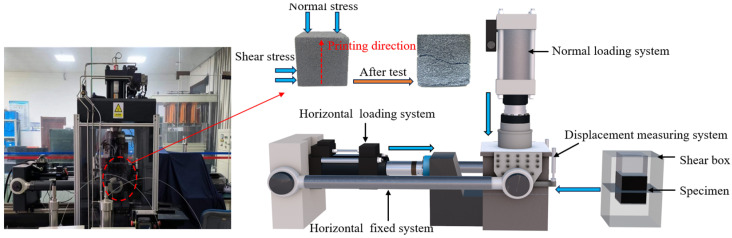
MTS816 rock mechanics shear test system and test process.

In this study, the BJT was used to prepare specimens of different sizes with an Easy3DP-S450 micro-droplet sand-powder printer (Wuhan, China) (see Figure 2a). During the manufacturing process, furan resin was used as the binder material. The chemical reaction between the binder and the curing agent (Wuhan, China) was utilized to bind the powder layer by layer from bottom to top, ultimately forming the entire specimen. This method offers advantages such as fast molding speed, short curing period, controllable strength, and customizable specimen sizes. The BJT specimen preparation process is illustrated in Figure 3. Before printing, the curing agent and sand powder were mixed evenly at a 6‰ ratio and then spread evenly in the feeding tank. A digital model was established in 3D software (Cinema 4D S24.035) and uploaded for printing. The printing steps are as follows: (1) the feeding tank is raised; (2) the powder feeding roller moves forward to evenly spread a layer of sand powder in the build tank; (3) the binder supply tank moves to the printing area, where the binder is sprayed by the nozzle and then the nozzle retracts from the printing area; (4) the build tank is lowered, and the above steps are repeated until the entire specimen is printed.

To ensure that the soft rock-like specimens prepared in this study have similar strength characteristics to the uniaxial compression specimens used in preliminary tests (the uniaxial compressive strength ranges between 14 and 18 MPa), the sand powder used had a particle size of 70–140 mesh (0.106–0.212 mm), and the ratio of curing agent to sand powder was 6‰. The powder layer thickness was set at 0.2 mm per layer. After printing, the specimens were naturally cured for 7 days, then sealed with plastic wrap and prepared for testing. In this study, cubic specimens with side lengths of 40 mm, 50 mm, 70 mm, and 100 mm were prepared, and compressive shear tests were conducted at three different shear velocities (Group A: 0.4 mm/min; Group B: 0.8 mm/min; Group C: 1.2 mm/min).

### 2.2. Shear Test Design

The shear test system is shown in Figure 3. The shear tests were conducted using a MTS816 rock mechanics shear testing machine (Eden Prairie, MN, USA). This instrument has a maximum axial compression force of 1459 KN, a maximum horizontal shear force of 261 KN, and a shear stroke of 100 mm. It features displacement loading with servo control and allows for real-time monitoring of both axial and shear displacements during the shear process.

During practical engineering, the shear behavior of rock masses may be influenced by different shear velocities. Variations in shear velocity can significantly affect the shear strength and failure patterns of the rock mass [33,34], a phenomenon known as the shear velocity effect. Studying the shear velocity effect (hereinafter referred to as the velocity effect) provides critical parameters for geotechnical engineering design, helps optimize support design and mining processes, and enhances safety and economic efficiency. Research by Tang et al. [35] and Huang et al. [11], among others, indicates that the impact of shear velocity on rock shear strength has not reached a consensus, with both velocity weakening (where shear strength is negatively correlated with shear velocity) and velocity strengthening (where shear strength is positively correlated with shear velocity) observed. Therefore, investigating the influence of different shear velocities on rock failure behavior is of significant importance. Based on this, the study conducted shear tests at three shear velocities (0.4 mm/min, 0.8 mm/min, and 1.2 mm/min) to explore the size effect of sand-powder 3DP soft rock under varying shear velocity conditions.

In order to prevent cracks or failures from occurring in the specimen during the application of normal stress, 3 MPa was chosen as the initial normal stress for conducting the shear test. Therefore, under normal stress of 3 MPa, shear tests were conducted on sand-powder 3DP soft rock specimens of different sizes at three shear velocities. The test grouping is shown in Table 1, where *ν* represents shear velocity, *S* represents specimen size, and *σ_n_* represents initial normal stress. The loading process is as follows: (i) The specimen is placed in the shear box of the testing machine. Initially, shear displacement is controlled by servo control at a velocity of 0.025 mm/min. Shearing force is applied until the shear device contacts the specimen and the shear force reaches 1 KN, at which point the application of shear force is stopped. (ii) Normal load is applied to the specimen using force control, increasing at a constant velocity of 0.2 MPa/s until reaching 3 MPa, after which it is maintained constant. (iii) Shear force is applied to the specimen at velocities of 0.4 mm/min, 0.8 mm/min, and 1.2 mm/min using displacement control. Data are recorded until the specimen loses its load-bearing capacity. The loading is then stopped, the specimen is removed, and its failure pattern is recorded.

## 3. Analysis of the Size Effect on Shear Characteristics of Sand-Powder 3D Printed Soft Rock

This section presents the results of shear tests conducted on sand-powder 3DP soft rock specimens of different sizes at three shear velocities. The shear stress–shear displacement curves were obtained and analyzed in conjunction with established patterns from research on the size effect in natural rocks. The analysis focused on how shear strength and shear deformation change with specimen size and shear velocity. The results reveal that the shear strength and shear deformation of sand-powder 3DP soft rock exhibit significant size and velocity effects, thereby validating the effectiveness of sand-powder 3DP soft rock in shear mechanics testing.

### 3.1. Analysis of Shear Mechanical Characteristics of Sand-Powder 3D Printed Soft Rock of Different Sizes

#### 3.1.1. Typical Shear Stress–Displacement Curve Characteristics

**Figure 4:**
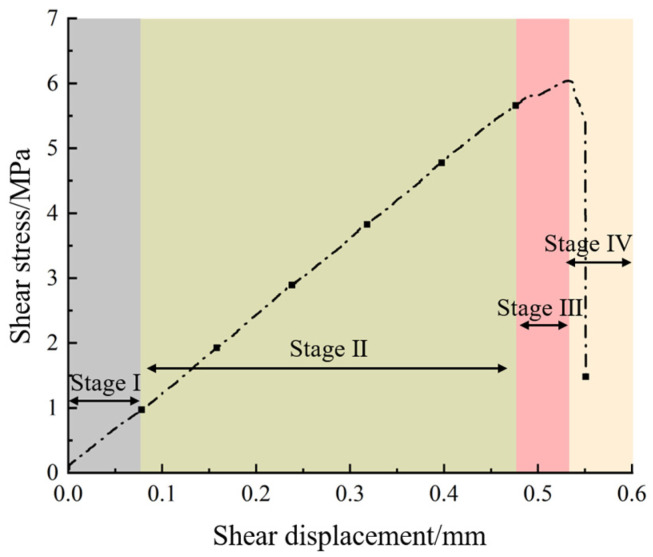
Shear stress–shear displacement curves of typical specimens.

The shear stress–displacement curve evolution of sand-powder 3DP soft rock specimens of different sizes is similar. For analysis, the 70 mm × 70 mm specimen at a shear velocity of 0.8 mm/min was selected as a representative specimen. The evolution process of the shear stress–shear displacement curve is as follows: As shown in Figure 4, the failure of sand-powder 3DP soft rock exhibits clear brittle characteristics, with the shear load on the curve rapidly dropping after reaching its peak. The specimen undergoes approximately four stages during shearing:

(1) Pre-peak compaction stage (Stage I): Under the action of shear force, microcracks within the specimen close, and broken particles gradually fill the internal pores, leading to an increase in effective stress. The curve exhibits a concave trend.
(2) Linear elastic stage (Stage II): The specimen undergoes elastic deformation due to the shear force, and the shear stress is proportional to the shear displacement, resulting in an approximately linear curve.
(3) Yield stage (Stage III): As the shear force continues to increase, cracks near the shear plane of the 3DP specimen initiate, propagate, and expand. The specimen loses its elasticity and exhibits plastic deformation, causing the curve to show a convex trend.
(4) Post-peak failure stage (Stage IV): After reaching the peak shear stress, the internal structure of the specimen can no longer withstand the shear force, leading to fracture and sliding failure along the shear plane. The shear stress decreases sharply, and the shear curve drops steeply.

Compared to the shear stress–displacement curves of natural rocks and conventional coal-like rocks [36,37], the initial compaction phase of the sand-powder 3DP soft rock in this study is not pronounced. This is because the sand-powder 3DP soft rock structure is already dense, and compaction has occurred in the specimen during the application of normal stress. As a result, when the experimental data collection begins, the curve does not show a distinct compaction phase.

#### 3.1.2. Analysis of the Variation Patterns of Size Effect and Velocity Effect

**Figure 5:**
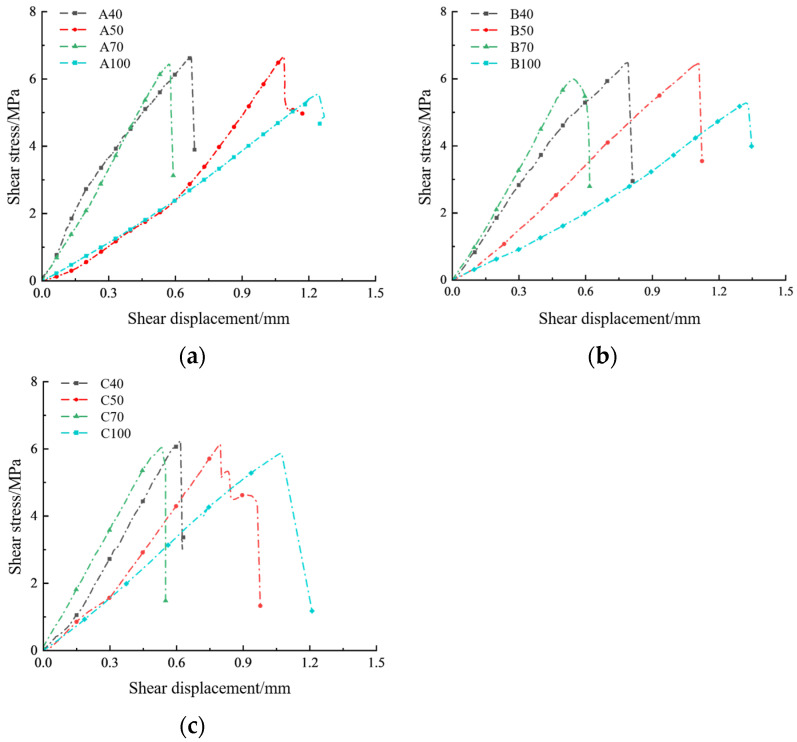
Curves of shear stress–shear displacement for sand-powder 3DP specimens at different shear velocities: (**a**) *ν* = 0.4 mm/min; (**b**) *ν* = 0.8 mm/min; (**c**) *ν* = 1.2 mm/min.

The results indicate that sand-powder 3DP soft rock exhibits significant size and velocity effects under different sizes and shear loading velocities. The shear stress–shear displacement curves for each group of specimens are shown in Figure 5. Under the same shear velocity, different-sized specimens show distinct size effects on shear strength, but the degree of this size effect varies with different shear velocities, specifically:

When *ν* = 0.4 mm/min (see Figure 5a): The shear strengths of the 40 mm (A40) and 50 mm (A50) specimens are relatively close, at 6.62 MPa and 6.60 MPa, respectively, with an increase in size resulting in only a 0.3% decrease in shear strength. When the specimen size increases to 70 mm (A70), the shear strength decreases to 6.44 MPa, representing a 2.7% reduction compared to 40 mm and a 2.4% reduction compared to 50 mm. For the 100 mm (A100) specimens, the shear strength further decreases to 5.55 MPa, showing a 15.9% reduction compared to 50 mm and a 13.8% reduction compared to 70 mm, indicating a more pronounced size effect compared to the previous sizes.

When ν = 0.8 mm/min (see Figure 5b): The shear strengths of the 40 mm (B40) and 50 mm (B50) specimens are 6.48 MPa and 6.45 MPa, respectively, with minimal impact from size variations on shear strength. The shear strength of the 70 mm (B70) specimen is 5.98 MPa, showing a 7.7% reduction compared to 40 mm and a 7.2% reduction compared to 50 mm. For the 100 mm (B100) specimens, the shear strength decreases to 5.27 MPa, representing an 11.9% reduction compared to 70 mm.

When *ν* = 1.2 mm/min (see Figure 5c): The results are similar to those at the other two shear velocities. The shear strength of the 50 mm (C50) specimens is 1.9% lower than that of the 40 mm (C40) specimens, and the 70 mm (C70) specimens show a 2.1% decrease compared to 50 mm. The 100 mm (C100) specimens exhibit a 2.5% reduction compared to 70 mm. Additionally, the shear stress–shear displacement curves reveal that with increasing specimen size, the post-failure section of the curve becomes more gradual. Larger specimens may also exhibit rebound behavior, as seen with A100.

**Figure 6:**
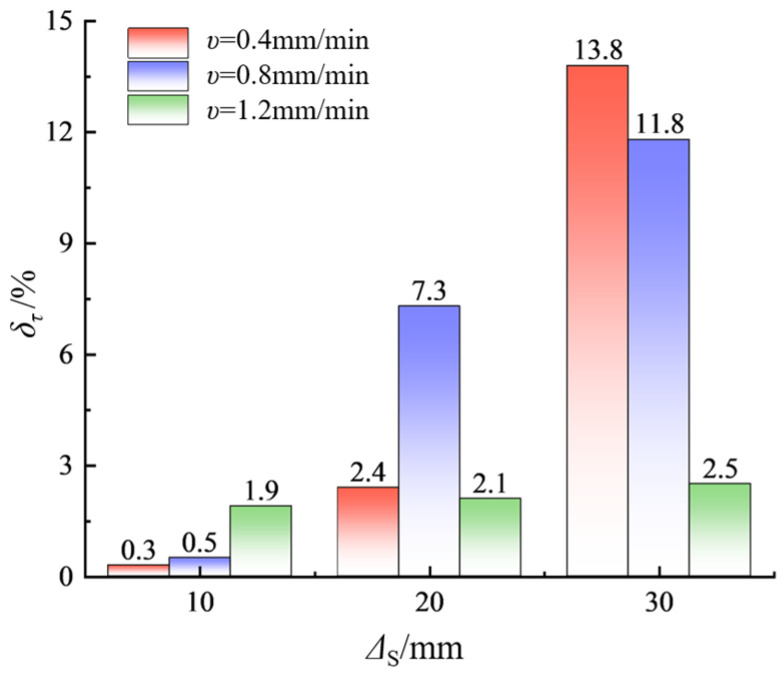
Bar chart of the relationship between percentage reduction in strength and incremental size increases.

To better illustrate the relationship between shear strength and specimen size, a quantitative analysis was conducted on the shear strength percentage decrease *δ_τ_* and the size increment gradient Δ*S* (the absolute value of the difference between adjacent sizes), as shown in Figure 6. From Figure 6, it can be observed that under the three different shear velocities, the reduction in shear strength is positively correlated with the size increment gradient. In other words, the degree of size effect on the shear strength of sand-powder 3DP soft rock is significant; the larger the size increment, the more the shear strength decreases. For example, when *ν* = 0.4 mm/min, an increase in size by 10 mm corresponds to a 0.3% reduction in shear strength. An increase of 20 mm results in a 2.4% reduction, and when the size increment gradient reaches 30 mm, the shear strength decreases by 13.8%.

**Figure 7:**
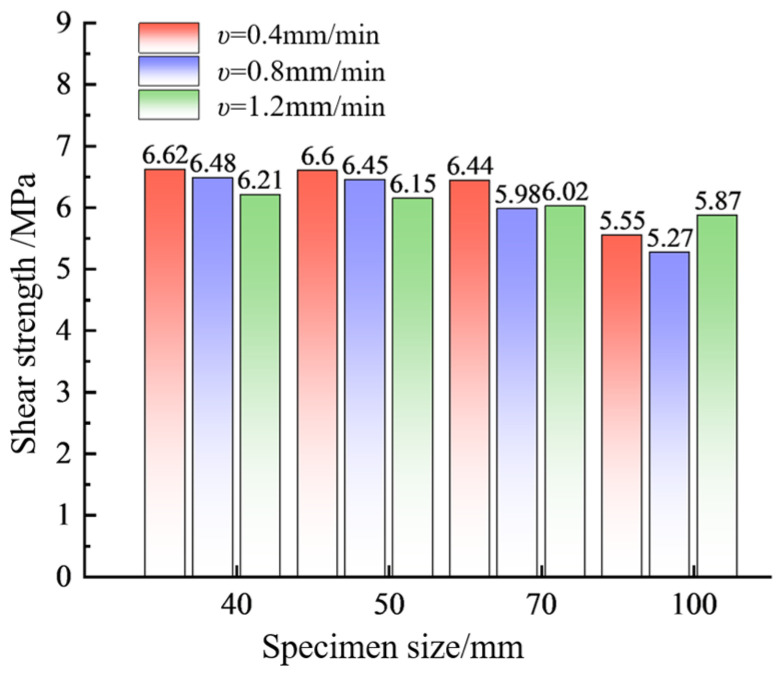
Bar chart of shear strength for sand-powder 3DP specimens at different shear velocities.

From Figure 7, it can be observed that specimens of the same size exhibit significant velocity dependence. Specifically, for specimen sizes of 40 mm and 50 mm, the shear strength decreases as the shear velocity increases [38]. Atapour [39] noted that increasing the shear velocity significantly reduces the shear contact area, leading to a decrease in strength. However, for specimen sizes of 70 mm and 100 mm, the shear strength initially decreases and then increases with the shear velocity. At lower shear velocities, the internal voids between particles are more effectively filled by the finer particles that result from fragmentation, leading to increased strength. At higher shear velocities, however, these finer particles do not fill the voids adequately, resulting in reduced shear strength. Moreover, increasing the shear velocity also restricts the size effect. At lower shear velocities, the difference in shear strength between different sizes is more pronounced, while at higher shear velocities, the shear strength tends to stabilize [40]. For example, at shear velocities of 0.4 mm/min and 0.8 mm/min, the increase in specimen size significantly reduces the shear strength, except for the 40 mm and 50 mm specimens, which are relatively close. When *ν* = 1.2 mm/min, the difference in shear strength between the four sizes is within 0.3 MPa.

In summary, the sand-powder 3DP soft rock used in the tests exhibits a significant size effect on shear strength. Specifically, under the same shear velocity and normal stress levels, the shear strength of the specimens decreases as the specimen size increases, and the reduction in shear strength is proportional to the increase in size gradient (the absolute value of the difference between adjacent sizes). Bazant [41] noted that larger specimens store more energy at the same stress level compared to smaller specimens, leading to earlier crack initiation in larger specimens and, consequently, lower shear strength. Additionally, the shear strength also shows a notable velocity effect. At lower shear velocities, the size effect is significant, with substantial differences in shear strength between specimens of different sizes. However, at higher shear velocities, the influence of specimen size on shear strength decreases, reducing the significance of the size effect. Furthermore, the shear failure of the sand-powder 3DP soft rock generally exhibits pronounced brittle characteristics, similar to the results obtained by Zhang et al. [37], Cheng et al. [42], and others using coal–rock combinations or sandstone specimens in shear tests.

### 3.2. Analysis of Shear Deformation Characteristics of Sand-Powder 3D Printed Soft Rock of Different Sizes

**Figure 8:**
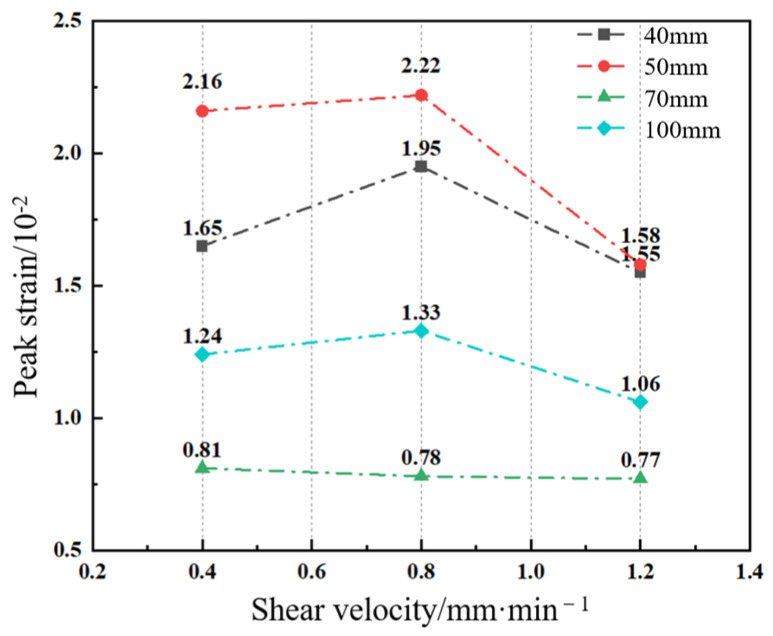
Curves of peak shear strain–shear velocity for sand-powder 3DP soft rock-like specimens of different sizes.

By comparing the peak shear strain values under different sizes and shear velocities (see Figure 8), it is evident that the peak shear strain of sand-powder 3DP soft rock exhibits a certain size effect, though no specific pattern is consistently observed. For 3DP specimens of the same size, the peak shear strain is not significantly affected by the shear velocity, generally showing a trend of increasing initially and then decreasing, with typical error values ranging from 0.02 to 0.5. However, when the shear velocity is fixed, the specimen size significantly influences the peak shear strain, as shown in Figure 8. This trend of initially increasing, then decreasing, and increasing again indicates that changes in specimen size affect shear strain, but there is no consistent linear relationship between the two.

**Figure 9:**
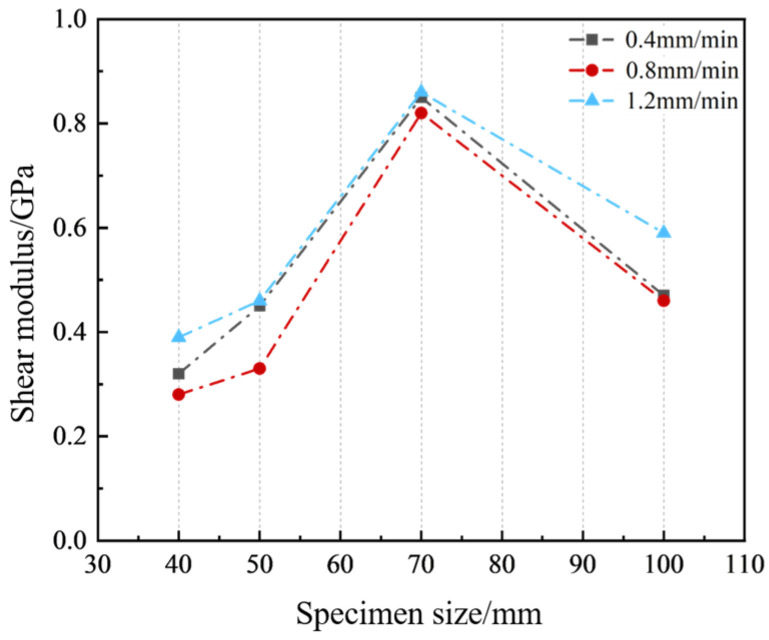
Curves of shear modulus and specimen size for sand-powder 3DP soft rock-like specimens of different sizes.

Figure 9 illustrates the variations in shear modulus for sand-powder 3DP soft rock specimens of different sizes under various shear velocities. The changes in shear modulus for different-sized specimens show a high degree of similarity. When the shear velocity is constant, the shear modulus of the specimens generally exhibits a trend of increasing initially and then decreasing with increasing specimen size. Among the different sizes, the shear modulus of the 70 mm specimens remains relatively consistent across all three shear velocities, with differences within 0.04 GPa. For other sizes, the shear modulus does not show significant changes with varying shear velocities, with differences within 0.1 GPa. From the above results, it is clear that the shear modulus of sand-powder 3DP soft rock specimens also exhibits significant size effects. However, changes in shear velocity have a minimal impact on the shear modulus, indicating a low correlation between the two factors.

In summary, the shear deformation characteristics of sand-powder 3DP soft rock show significant size effects. In terms of shear strain, the shear strain values change significantly with increasing specimen size, and this variation is not influenced by changes in shear velocity. There is no consistent linear relationship between shear strain and specimen size; instead, shear strain exhibits a nonlinear trend, increasing, then decreasing, and increasing again with specimen size. Similarly, different sizes of sand-powder 3DP soft rock exhibit significant size effects on shear modulus, although changes in shear modulus due to varying shear velocities are not significant, especially when the specimen size is 70 mm, where the impact of shear velocity variations can be considered negligible.

## 4. Analysis of Size Effects on Shear Failure Characteristics in 3D Printed Soft Rock

This section provides a detailed analysis of the failure patterns of sand-powder 3DP soft rock specimens of different sizes after shear tests. By classifying the failure patterns of the shear section after failure, and based on this, the binary image processing of the shear failure surface is carried out, and the influence of size on the shear failure characteristics of sand-powder 3DP soft rock is discussed. By analyzing the degree of wear on the failure surfaces, the specific effects of specimen size on shear failure characteristics are revealed.

### 4.1. Analysis of Size Effect on Shear Failure Pattern in 3D Printed Soft Rock

**Figure 10:**
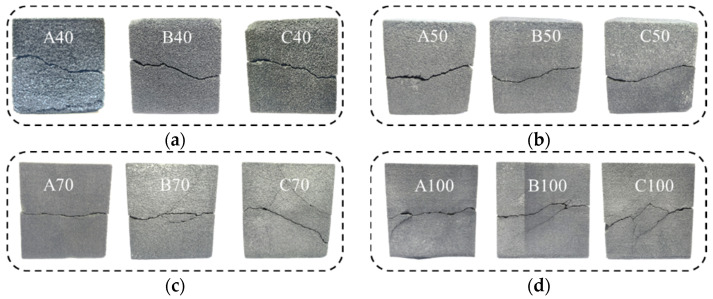
Shear failure patterns of sand-powder 3DP soft rock-like specimens with different sizes: (**a**) specimen size = 40 mm; (**b**) specimen size = 50 mm; (**c**) specimen size = 70 mm; (**d**) specimen size = 100 mm.

Based on the experimental data obtained under three different shear velocities, a comparative analysis of the shear failure characteristics of specimens of various sizes (see Figure 10) was conducted. The results indicate a high degree of consistency in the failure patterns of specimens of the same size under different shear velocities. Specifically, in specimens of smaller sizes (e.g., 40 mm and 50 mm), the shear failure surfaces were observed to be relatively intact, with only the primary shear cracks present and no secondary cracks forming. However, in larger specimens (70 mm and 100 mm), the emergence of multiple secondary cracks was significantly more pronounced. Bazant [41], from the perspective of fracture energy, posits that in larger specimens, the stored energy is higher than in smaller specimens. Consequently, larger specimens exhibit earlier crack initiation, are more susceptible to failure, and generate a greater number of cracks.

**Figure 11:**
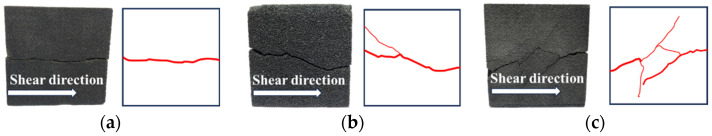
Three shear failure patterns of sand-powder 3DP soft rock-like specimens: (**a**) planar failure; (**b**) step-like failure; (**c**) cross-like failure.

Furthermore, based on the analysis of photos and sketches of the different specimens after failure (see Figure 11), the shear failure patterns on the surfaces can be categorized into three basic types:

(1) Planar failure: As shown in Figure 11a, the shear failure surface of the specimen extends in a direction that is generally parallel to the shear direction. This type of failure often occurs at shear velocities of 0.4 mm/min and 0.8 mm/min, such as in specimens A70, B70, and A100. When the shear velocity is low, the shear force cannot overcome the internal strength and cohesion of the material, causing the specimen to slide along internal planes of weakness, resulting in a shear failure surface that is nearly parallel to the shear direction.
(2) Step-like failure: As shown in Figure 11b, the shear failure surface exhibits a stepped, undulating pattern. Two cracks formed at the boundary of the specimen are not on the same horizontal plane but are instead inclined, creating a stepped crack in the middle of the specimen. This type of failure is not influenced by shear velocity and occurs in specimens with dimensions of 40 mm and 50 mm.
(3) Cross-like failure: As shown in Figure 11c, the specimen develops two vertical cracks that do not directly connect but intersect and offset in the middle of the specimen, leading to a cross-like failure. This failure pattern commonly occurs in larger specimens, such as C70, B100, and C100. Larger specimens accumulate more energy than smaller ones, which can trigger the formation and propagation of cracks. These micro-cracks prevent the specimen from experiencing sliding failure along the shear direction or vertical stepped penetration, ultimately resulting in a cross-like failure pattern.

The analysis of the shear failure patterns of the specimens reveals that the shear failure patterns of the sand-powder 3DP soft rock are influenced by both specimen size and shear velocity. However, no strong correlation with a specific factor is observed, meaning that no definitive conclusion can be drawn regarding the occurrence of a particular failure type under certain sizes or shear velocities. Nevertheless, the analysis of the extent of failure indicates that larger sand-powder 3DP soft rock specimens are more likely to develop secondary cracks. The presence of these secondary cracks further results in lower shear strength for larger specimens compared to smaller ones.

### 4.2. Analysis of Size Effect on Shear Wear Characteristics in 3D Printed Soft Rock

**Figure 12:**
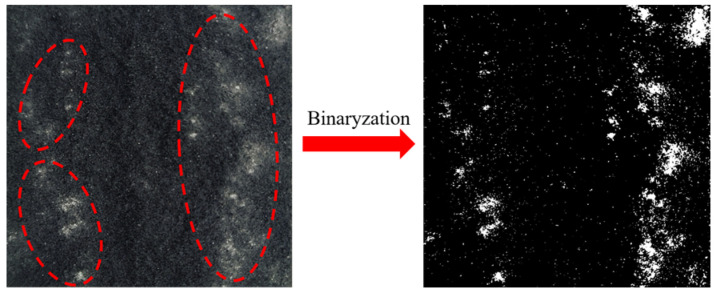
Diagram of the shear failure surface of the specimen.

To better illustrate the size effect of shear failure in sand-powder 3DP soft rocks, photographs of the shear failure surfaces after testing were taken, as shown in Figure 12. The areas highlighted with red dashed lines indicate regions where shear wear occurred during the shear process. Photographs of the failure surfaces from different specimens were processed with binary image analysis using Matlab (R2019a).

**Figure 13:**
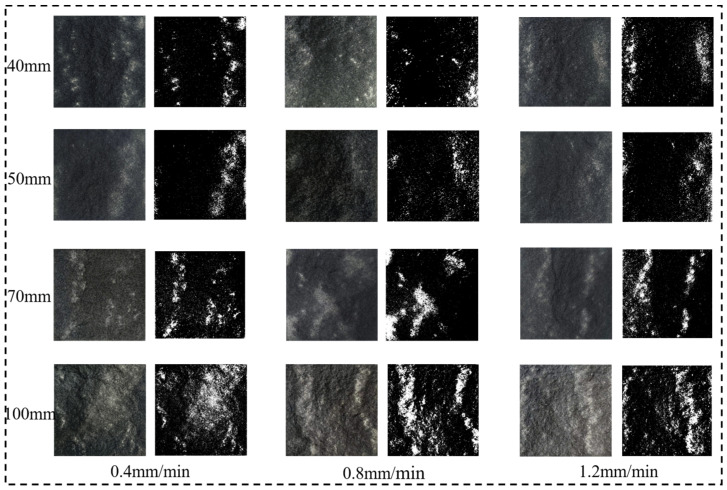
Shear wear conditions and binarized images of specimens of different sizes.

Figure 13 illustrates the wear patterns and binary images of shear failure surfaces for sand-powder 3DP specimens under different shear velocities. In the binary images, black areas indicate regions with minimal wear, while white areas represent regions with significant wear. It is evident from Figure 13 that for specimens of the same size, the wear patterns on the shear failure surfaces are quite similar regardless of the shear velocity. Comparing the wear distribution across four different sizes, it is observed that most wear occurs along the edges of the shear failure surface. This is consistent with the failure pattern of the specimens, where fractures initiate from the edges during shear, leading to significant wear on the sides of the shear plane. As cracks propagate toward the center of the specimen, the top and bottom sections of the specimen shift relative to each other under the shear force, resulting in less wear in the middle of the shear surface. Additionally, it is noticeable that as the specimen size increases, the degree of wear on the shear failure surface also intensifies.

Dieterich [40] suggests that the surface friction of rock shear failure is related to the shear velocity. To better illustrate the variation of wear conditions on the shear failure surface with changes in specimen size and shear velocity, the proportion of the wear area to the total area (referred to as the wear area ratio, *λ*) was calculated based on binarized images:

(1) $$\lambda =\frac{S_{w}}{S_{w}+S_{b}}$$

where *S*_w_ is the area of white pixels after binary segmentation, and *S*_b_ is the area of black pixels after binary segmentation.

Additionally, the increase rate of the wear area ratio (Δ*λ_υ_*) for adjacent shear velocities was statistically analyzed. As shown in Table 2, for specimens of the same size, both *λ* and Δ*λ_υ_* significantly increase with the increase in shear velocity. Notably, the Δ*λ_υ_* from 0.8 mm/min to 1.2 mm/min is significantly higher than the previous gradient. However, Δ*λ_υ_* does not exhibit a consistent pattern with the increase in specimen size, indicating that the wear area ratio induced by the shear velocity is not influenced by specimen size variation. In stark contrast, under the same shear velocity, the value of *λ* significantly increases with an increase in specimen size. By averaging the *λ* values for the same size (*Mλ_s_*) and calculating the increase rate of the mean wear area ratio (Δ*λ_s_*), it is evident from the data in Table 2 that Δ*λ_s_* exhibits significant size dependence. Furthermore, the increase in Δ*λ_s_* corresponds closely to the decrease in shear strength, with both showing greater increases with larger size gradients.

In summary, the extent of wear on the shear surface, as one of the characteristics of rock shear failure, exhibits a size effect similar to that of shear strength. When the shear velocity is higher, the wear on the shear surface increases, resulting in reduced shear strength of the specimen. Additionally, the rate of increase in the wear area on the shear surface is proportional to the rate of decrease in shear strength with respect to the specimen size gradient.

## 5. Discussion

**Figure 14:**
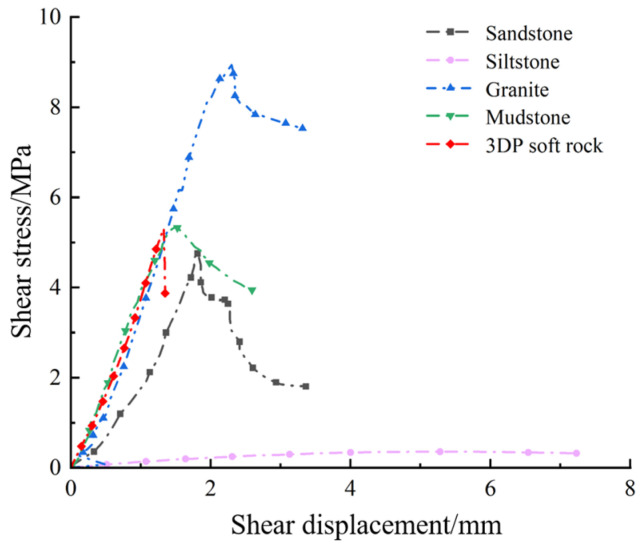
Shear stress–shear displacement curves of sand powder 3DP rock-like and natural rocks.

Both in practical engineering and laboratory research, exploring the size effect and velocity effect of rocks is crucial. Recent studies by scholars both domestically and internationally have shown that sand-powder 3DP technology produces soft rock-like materials with properties similar to natural rocks, such as comparable elastic–plastic mechanical characteristics, the ability to create complex defects and stable characteristics. The results of this study further indicate that sand-powder 3DP soft rocks exhibit shear mechanical properties similar to those of natural rocks. As shown in Figure 14, the shear stress–shear displacement curves obtained in this study are similar to those of natural sandstone and mudstone under the same testing conditions. The shear strength of 3DP soft rocks is comparable to certain mudstone and sandstone, stronger than some siltstone, but significantly lower than granite. Regarding peak displacement, 3DP soft rocks exhibit lower values compared to other natural rocks. The study suggests that the reduced peak displacement of sand-powder 3DP rock-like materials is related to the angle between the loading direction and the printing direction [43]. The research found that both changes in specimen size and shear velocity affect the shear mechanical properties of the specimens. The phenomenon where the shear strength of specimens decreases with increasing size is similar to the results obtained by Barton et al. [44] using natural sandstone in direct shear tests and is consistent with the findings of Huang [38] using synthetic soft rocks. This conclusion supports the continued use of powder bonding technology (BJT) to prepare sand-powder 3DP soft rocks for studying rock shear characteristics and size effects. As shown in Figure 14, the strength of the rock-like specimens produced through sand-powder 3D printing in this study is relatively low. Recent research has indicated that incorporating nanocomposites into the printing materials can significantly improve the performance of the printed samples [45]. For instance, Jiang et al. [46] demonstrated that the inclusion of glass fibers in the sand-powder mix led to a 1.5-fold increase in the uniaxial compressive strength of the sand-powder 3D printed specimens.

This study found that for specimen sizes of 70 mm and 100 mm, the shear strength initially decreases and then increases with increasing shear velocity. This result is inconsistent with the findings of Zheng [47] and Atapour [39], who observed a decrease in shear strength with increasing shear velocity for large specimens. Chai [33] suggested that this discrepancy might be related to the filling of internal voids by fine particles during failure. Additionally, due to limitations in test conditions, this study did not investigate a range of shear velocities, so further research and discussion are needed to better understand the mechanisms underlying the changes in shear characteristics of sand-powder 3DP soft rocks due to shear velocity variations.

On the other hand, rock masses in practical engineering generally exist on large scales, whereas laboratory studies are constrained to small-scale specimens due to test limitations. The study of shear characteristics of soft rocks at multiple scales in this research provides insights into selecting appropriate sizes for more complex shear tests. With the advantages of sand-powder 3D printing technology in rapidly preparing specimens with embedded fracture networks, future research could focus on 3DP specimens of specific sizes that closely resemble the physical properties of natural engineering rock masses to study fractured rock masses under various geological conditions. In this study, all specimens were fabricated using identical printing parameters. However, for the BJT method, it is possible to simulate different types of natural rocks by adjusting the printing parameters, thereby facilitating the investigation of various categories of 3D-printed rock-like materials. In this study, all tests were conducted with the printing direction perpendicular to the shear direction. However, related research indicates that different loading directions can have varying effects on the test results [48,49,50]. Therefore, to better apply sand-powder 3D printing technology in rock mechanics experimental research, future studies could investigate the effects of different angles between the printing direction and shear direction. Additionally, due to the limitation of test conditions in this study, shear tests were only conducted under constant normal load (CNL) conditions. However, in deep engineering, rocks are more often in a constant normal stiffness (CNS) condition. To enhance the applicability of sand-powder 3DP soft rocks in shear mechanics research, further studies should be conducted under different boundary conditions to better serve rock mechanics engineering.

## 6. Conclusions

This study utilized sand-powder 3D printing technology to fabricate rock specimens of different sizes. Through typical shear tests, the effects of specimen size and shear velocity on the strength characteristics, deformation characteristics, and failure modes of the specimens were investigated. The specific conclusions are as follows:

(1) The shear strength decreases progressively with increasing specimen size, and the reduction in shear strength is proportional to the increase in size gradient. Additionally, the increase in shear velocity shows a suppressive effect on the significance of the size effect. When the shear velocity is 1.2 mm/min, the difference in shear strength among different-sized specimens is within 0.3 MPa.
(2) The peak shear strain is significantly affected by specimen size but does not exhibit a linear relationship. For specimens of the same size, as the shear velocity increases, the peak shear strain initially increases and then decreases, with differences generally within 0.5 × 10^−2^, indicating that shear strain is not significantly influenced by shear velocity.
(3) For specimens of the same size, the failure patterns under different shear velocities can be categorized into three types: planar failure, step-like failure, and failure. However, these failure patterns do not show a consistent size effect pattern. The degree of failure also exhibits a certain size correlation, with larger specimens more prone to secondary cracks and a significant increase in failure degree. However, no significant shear velocity correlation is observed.
(4) The wear on shear failure surfaces exhibits a certain size effect. As the specimen size increases, both the degree of wear and the wear area increase. The wear area ratio and the rate of increase in wear area ratio show certain size effects and shear velocity effects.

In summary, the reliable results obtained from uniformly stable samples in this study enhance our understanding of the shear size effect in rocks, supporting the notion of a negative size effect. The study also found that increasing the shear velocity significantly reduces the extent of the size effect, indicating that at sufficiently high shear velocities, the mechanical property differences due to varying sizes diminish. This finding is crucial for optimizing engineering design parameters, improving predictive models, enhancing construction techniques, and bolstering risk assessments. By adjusting construction speed and methods to control the shear velocity, potential issues arising from the shear size effect can be mitigated. Additionally, the analysis of failure patterns aids in predicting the extent of shear failure in actual rock engineering. These conclusions further demonstrate the significant potential of sand-powder 3DP in rock mechanics research. The effectiveness of sand-powder 3DP soft rocks in the physical simulation of rock shear mechanics has been validated, laying the foundation for future studies on rock masses with complex fractures.

## Figures and Tables

**Table 1:** Test parameters.

Specimen Number	Shear Velocity *υ*/mm/min	Specimen Size *S*/mm	Normal Stress *σ_n_*/MPa
A40	0.4	40	3
A50		50	
A70		70	
A100		100	
B40	0.8	40	
B50		50	
B70		70	
B100		100	
C40	1.2	40	
C50		50	
C70		70	
C100		100	

**Table 2:** Shear failure surface wear area ratio under different test parameters.

Specimen Size *S*/mm	Shear Velocity *υ*/mm/min	*λ*	Δ*λ_υ_*	*Mλ_s_*	Δ*λ_s_*
40	0.4	0.051		0.055	
	0.8	0.053	3.9%		
	1.2	0.061	15.1%		5.5%
50	0.4	0.058		0.058	
	0.8	0.060	3.5%		
	1.2	0.063	5%		59%
70	0.4	0.087		0.092	
	0.8	0.088	1.1%		
	1.2	0.102	15.9%		100%
100	0.4	0.182		0.184	
	0.8	0.184	1.1%		
	1.2	0.187	1.6%		

## Data Availability

The original contributions presented in this study are included in the article. Further inquiries can be directed to the corresponding author.
